# High-Dose Antipsychotic Therapy Monitoring in a Community Rehabilitation Cohort: A Cross-Sectional Audit From Sussex Partnership NHS Foundation Trust

**DOI:** 10.1192/bjo.2026.11724

**Published:** 2026-06-30

**Authors:** Haneen Eltayeb, Bilal Ahmad

**Affiliations:** Sussex Partnership NHS Foundation Trust, Eastbourne, United Kingdom

## Abstract

**Aims:**

High-Dose Antipsychotic Therapy (HDAT) is associated with increased risk of adverse physical health outcomes and requires robust monitoring in line with Royal College of Psychiatrists (RCPsych) guidance and local Trust policy. This audit aimed to:

1. Assess compliance with Sussex Partnership NHS Foundation Trust (SPFT) HDAT physical health monitoring policy.

2. Assess compliance with theRCPsychconsensus statement on HDAT monitoring.

3. Identify patterns of antipsychotic polypharmacy within a community rehabilitation cohort.

**Methods:**

A cross-sectional audit was conducted in December 2025 within the East Sussex Rehabilitation Pathway (ESRP), part of SPFT. The cohort included adults aged 18–65 receiving care from a community rehabilitation team in Eastbourne. Medication records were reviewed, and the antipsychotic high-dose calculator was used to identify patients prescribed HDAT in accordance with BNF criteria. Data were collected from electronic patient records (Carenotes) and HDAT monitoring forms. Variables included diagnosis, ethnicity, number of antipsychotics prescribed, and completion of recommended HDAT monitoring (blood tests, ECG, weight/physical observations, GASS side-effect scale, and HDAT documentation).

**Results:**

Eleven patients met criteria for HDAT, representing approximately 18% of the rehabilitation caseload. The majority were White British (64%).

Paranoid schizophrenia was the most common diagnosis (64%), followed by schizo affective disorder (27%) and persistent delusional disorder (9%). Most patients (73%) were prescribed two antipsychotics, while 27% were prescribed three, indicating a high prevalence of antipsychotic polypharmacy.

Compliance with HDAT monitoring was variable: blood tests and weight/physical observations were completed in 45% of patients, ECGs in 27%, GASS was offered in 27%, and HDAT documentation was fully up to date in 36% of cases.

**Conclusion:**

HDAT use within this community rehabilitation cohort was relatively common and largely driven by antipsychotic polypharmacy in patients with severe and enduring psychotic illness. Monitoring compliance was inconsistent and fell short of RCPsych and local policy standards. These findings highlight the need for improved identification of HDAT, better utilisation of monitoring tools, and strengthened multidisciplinary oversight to ensure safe, high-quality care. Findings were presented to the multidisciplinary team, and an action plan for improvement was formulated, with re-audit planned in six months.

